# Identification and causes of metabonomic difference between orthotopic and subcutaneous xenograft of pancreatic cancer

**DOI:** 10.18632/oncotarget.18057

**Published:** 2017-05-22

**Authors:** Bohan Zhan, Shi Wen, Jie Lu, Guiping Shen, Xianchao Lin, Jianghua Feng, Heguang Huang

**Affiliations:** ^1^ Department of Electronic Science, Fujian Provincial Key Laboratory of Plasma and Magnetic Resonance, Xiamen University, Xiamen 361005, China; ^2^ Department of General Surgery, Fujian Medical University Union Hospital, Fuzhou 350001, China

**Keywords:** pancreatic ductal adenocarcinoma, xenograft models, nuclear magnetic resonance, metabonomics

## Abstract

Pancreatic ductal adenocarcinoma (PDAC) is one of the most lethal tumors. However, the methodological differences between orthotopic and subcutaneous xenograft (OX and SX) models will cause confusion in understanding its pathological mechanism and clinical relevance. In this study, SX and OX models were established by implanting Panc-1 and BxPC-3 cell strains under skin and on the pancreas of mice, respectively. The tumor tissue and serum samples were collected for^1^H NMR spectroscopy followed by univariate and multivariate statistical analyses. As results, no obvious metabonomic difference was demonstrated in serum between the two models, however, the model- and cell strain-specific metabonomic differences were observed in tumor tissues. According to the KEGG analysis, ABC transporters, glycerophospholipid metabolism, purine metabolism and central carbon metabolism were identified to be the most significant components involved in metabonomic differences. Considering the methodological discrepancy in SX and OX models, such differences should be contributed to tumor microenvironment. In general, SX are not equivalent to OX models at molecular level. Subcutaneous transplantation displayed its inherent limitations though it offered a simple, inexpensive, reproducible and quantifiable advantage. And orthotopic transplantation may be favorable to simulate PDAC in patients due to its similar pathogenesis to human pancreatic cancer.

## INTRODUCTION

Pancreatic ductal adenocarcinoma (PDAC) is an extremely lethal malignancy with a devastated prognosis whose five-year survival rate is less than 7% [1]. Most patients are diagnosed in late stage and resectable rate of the tumors is only 20%. This situation makes it almost impossible to observe the whole process of tumor genesis and development in clinical work, and therefore understanding of its pathological mechanism mainly depended on animal models. In past decades, scientists have exerted great efforts to establish various animal models for PDAC, which can be served as a cornerstone for establishing favorable platforms for the clinical diagnosis and therapy of PDAC patients [2, 3].

Among animal models, transplantation models, including subcutaneous xenograft (SX) and orthotopic xenograft (OX) models, are most widely used to investigate pathology, diagnosis and therapy for cancers. As highly advocated by researchers, OX models of carcinoma can simulate the growing environment and enable them to maintain the features of the primary tumor, thus being more patient-like models [4]. Furthermore, in some specific tumors like gallbladder cancer, the ascites generation, lymph node and liver metastasis can only be observed in OX models but not in SX models [5], indicating that OX models is superior to SX models. However, SX models are still widely used in scientific researches especially for pharmaceutical trails due to more facilitation of their establishing, monitoring and operating than OX models. Nowadays, in the face of the popularity of system biological studies including genomics, transcriptomics, proteomics especially metabonomics, there is an increasing demand to compare applicability of these two models at molecular level. Therefore, considering that most of previous researches focus on the differences of tumorous biological behaviors while the metabonomic differences generated by the methodological diversity of modeling are still unknown, we tried to evaluate the pros and cons of these two models in PDAC and get their metabonomic difference between OX and SX models by using NMR-based metabonomics methods, and further serve scientific researches like pathological and pharmaceutical experiments.

## RESULTS

### Metabolic profiles of serum and tissues from mice with PDAC

**Figure 1:**
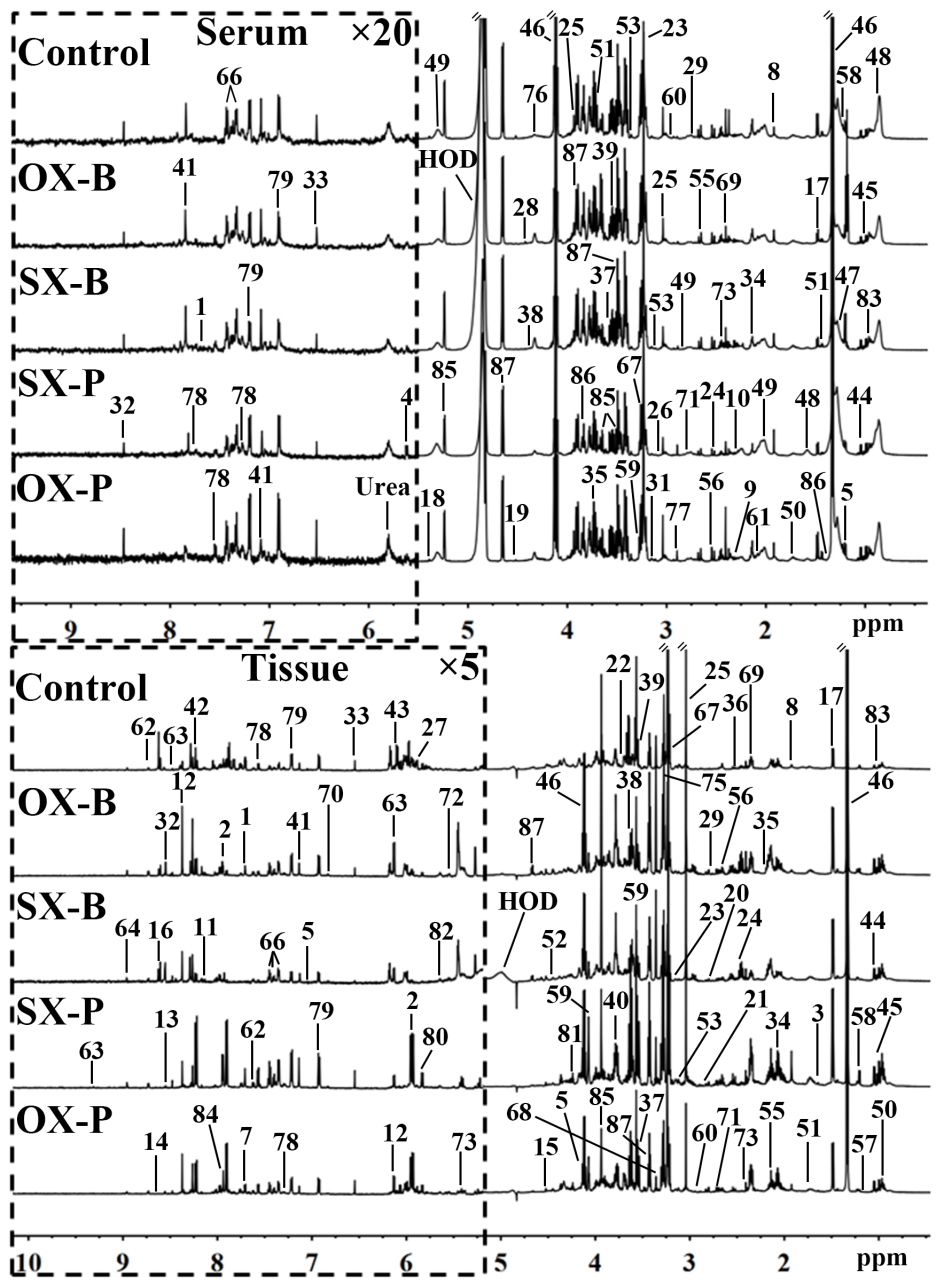
Representative 600 MHz ^1^H NMR spectra of the serum (top panel) and tissue (bottom panel) samples from control, orthotopic and subcutaneous xenograft (OX and SX) mouse models induced by Panc-1 (-P) and BxPC-3 (-B) cell strains The spectral regions in the dashed boxes were magnified 20 (for serum) and 5 (for tissue) times for the purpose of clarity. Keys for the assignments of peaks were showed in Table 1.

^1^H NMR spectra of serum and tissue provide the corresponding metabolic profiles of the mice. The characteristic metabonomic profiles of serum and tissue from SX and OX models induced by BxPC-3 and Panc-1 cells were demonstrated in the corresponding NMR spectra (Figure 1). The resonance assignments were performed based on the published articles [6, 7, 8], public [9] and in-house developed databases, and the corresponding metabolites were marked in NMR spectra in the form of serial numbers, which can be viewed in Table 1 for details. All xenograft models demonstrated the similar metabolic profiles in serum metabonomes especially those induced by the same pancreatic cancer cell line although some characterized metabolites could be identified from the controls such as the higher LDL and pyruvate concentrations and the lower glycerophosphorylcholine (GPC) concentrations in xenograft models than in controls (Top panel in Figure 1). Tissue metabonomes of the xenograft models showed the obviously different metabolic profiles from those of the controls (Bottom panel in Figure 1). Furthermore, orthotopic xenograft demonstrated distinct metabonomic variations from subcutaneous xenograft, and Panc-1 cell strain induced more obvious metabolic differences than BxPC-3 between OX and SX groups. However, the visual comparison cannot provide the detailed biological information for assessment of similarities and differences between SX and OX models, thus, multivariate statistical analysis could be helpful for such specific bio-information.

**Table 1:** List of metabolites identified from NMR spectra of serum and tissue and the corresponding assignments

No.	Abbr.	Metabolites	^1^H chemical shift (ppm)(multiplicity)	Sample
1	1-MH	1-Methylhistidine	7.08(s^a^), 7.69(s)	S/T^b^
2	DU	2-Deoxyuridine	5.95(d), 7.87(d)	T
3	2-HB	2-Hydroxybutyrate	0.90(t), 1.70(m)	T
4	MBC	2-Methylbutyroylcarnitine	5.61(dd)	S
5	3-HB	3-Hydroxybutyrate	1.20(d), 2.31(dd), 2.41(dd), 4.16(m)	S/T
6	3-MH	3-Methylhistinine	7.03(s), 7.90(s)	T
7	MC	5-Methylcytidine	7.69(d)	T
8	Ace	Acetate	1.92(s)	S/T
9	AA	Acetoacetate	2.28(s)	S
10	Act	Acetone	2.23(s)	S
11	Ad	Adenine	8.11(s), 8.12(s)	T
12	Ade	Adenosine	4.29(dd), 4.44(dd), 6.11(d), 8.37(s)	T
13	ADP	Adenosine diphosphate	8.55(s)	T
14	PAP	Adenosine 3′,5′-diphosphate	8.59(s)	T
15	AMP	Adenosine monophosphate	4.52(d), 6.15(d), 8.27(s), 8.61(s)	T
16	ATP	Adenosine triphosphate	8.53(s)	T
17	Ala	Alanine	1.48(d)	S/T
18	All	Allantoin	5.39(s)	S
19	Asc	Ascorbate	4.52(d)	S
20	Asn	Asparagine	2.88(dd), 2.95(dd), 3.99(dd)	T
21	Asp	Aspartate	2.70(dd), 2.81(dd), 3.94(dd)	T
22	Bet	Betaine	3.27(s), 3.91(s)	T
23	Cho	Choline	3.21(s)	S/T
24	Ci	Citrate	2.53(d), 2.67(d),	S/T
25	Cr	Creatine	3.04(s), 3.93(s)	S/T
26	Cn	Creatinine	3.05(s), 4.06(s)	S
27	Cyd	Cytidine	5.87(d), 7.81(d)	T
28	DHA	Dihydroxyacetone	4.42(s)	S
29	DMA	Dimethylamine	2.72(s)	S/T
30	Eth	Ethanol	1.18(t), 3.61(q)	S
31	EA	Ethanolamine	3.15(t), 3.86(t)	S/T
32	For	Formate	8.46(s)	S/T
33	Fum	Fumarate	6.52(s)	S/T
34	Glu	Glutamate	2.08(m), 2.12(m), 2.35(m), 3.78(t)	S/T
35	Gln	Glutamine	2.14(m), 2.45(m), 3.78(t)	S/T
36	GSH	Glutathione	2.56(m), 2.99(m)	T
37	G	Glycerol	3.56(dd ), 3.66(dd), 3.80(m)	S/T
38	GPC	Glycerophosphorylcholine	3.23(s), 3.68(m), 4.33(m)	S/T
39	Gly	Glycine	3.57(s)	S/T
40	GA	Guanidoacetate	3.80(s)	T
41	His	Histidine	7.05(s), 7.82(s)	S/T
42	HX	Hypoxanthine	8.20(s), 8.22(s)	T
43	Ino	Inosine	4.26(dd), 6.08(d), 8.25(s), 8.34(s)	T
44	IB	Isobutyrate	1.07(d)	S/T
45	Ile	Isoleucine	0.94(t), 1.01(d), 1.26(m)	S/T
46	Lac	Lactate	1.33(d), 4.11(q)	S/T
47	LDL	Low density lipoprotein	0.86(br), 1.28(br)	S
48	VLDL	Very low density lipoprotein	0.89(br), 1.30(br), 1.58(br)	S
49	L	Lipid	2.01(br), 2.23(br), 2.78(br), 5.31(br)	S
50	Leu	Leucine	0.96(t),1.70(m)	S/T
51	Lys	Lysine	1.46(m), 1.73(m), 1.91(m), 3.01(m), 3.76(t)	S/T
52	Mal	Malate	4.31(dd)	T
53	M	Malonate	3.12(s)	S/T
54	Mol	Methanol	3.36(s)	S
55	Met	Methionine	2.14(s), 2.65(t)	S/T
56	MA	Methylamine	2.61(s)	S/T
57	MIB	Methylisobutyrate	1.18(d)	T
58	MM	Methylmalonate	1.22(d), 3.12(q)	S/T
59	m-I	*myo*-Inositol	3.27(t), 3.56(dd), 3.62(t), 4.07(t)	S
60	DMG	N,N-Dimethylglycine	2.93(s)	S/T
61	NAG	N-Acetyl-glycoprotein signals	2.04(s)	S
62	NA	Nicotinamide	7.60(dd), 8.25(dd), 8.72(dd), 8.94(d)	T
63	NAD	Nicotinamide adenine dinucleotide	4.37(m), 4.42(m), 4.49(m), 4.51(m), 6.04(d), 6.09(d), 6.12(d), 8.14(s), 8.42(s), 8.83(d), 9.14(d), 9.34(d)	T
64	NMA	N-Methylnicotinamide	4.49(s), 8.90(d), 8.98(d), 9.30(s)	T
65	Pan	Pantothenate	0.84(s), 0.90(s)	T
66	Phe	Phenylalanine	4.00(m), 7.33(d), 7.37(t), 7.43(m)	S/T
67	PC	Phosphocholine	3.22(s), 4.18(m)	S/T
68	Pro	Proline	3.33(m)	T
69	Py	Pyruvate	2.39(s)	S/T
70	Qu	Quinone	6.81(s)	T
71	Sar	Sarcosine	2.72(s), 3.60(s)	S/T
72	Sph	Sphignosine	5.52(dd), 5.56(dd), 5.74(dd)	T
73	Sur	Surcose	5.43(s)	T
74	Suc	Succinate	2.41(s)	S/T
75	Tau	Taurine	3.27(t), 3.43(t)	T
76	Thr	Threonine	1.33(d), 3.59(dd), 4.26(m)	S
77	TMA	Trimethylamine	2.89(s)	S
78	Trp	Tryptophan	7.28(m), 7.30(s), 7.53(d), 7.73(d)	S/T
79	Tyr	Tyrosine	6.91(d), 7.20(d)	S/T
80	Ura	Uracil	5.80(d), 7.55(d)	T
81	Ud	Uridine	4.24(t), 4.36(t), 5.91(d), 7.89(d)	T
82	UDG	Uridinediphosphate glucose	5.61(dd), 5.97(m), 7.96(d)	T
83	Val	Valine	0.99(d), 1.04(d), 2.28(m)	S/T
84	Xan	Xanthine	7.91(s)	T
85	α-Glc	α-Glucose	3.42(t), 3.54(dd), 3.71(t), 3.73(t), 3.84(m), 5.24(d)	S/T
86	HIV	α-Hydroxyisovalerate	1.36(s)	S
87	β-Glc	β-Glucose	3.25(dd), 3.41(t), 3.46(dd), 3.49(t), 3.90(dd), 4.65(d)	S/T

^a^Multiplicity: s: singlet; d: doublet; t: triplet; q: quartet; dd: doublet of doublets; m: multiplet; br: broad resonance

^b^S: serum; T: tissue.

### Metabolic characteristics of SX and OX models in serum metabonomes

**Figure 2:**
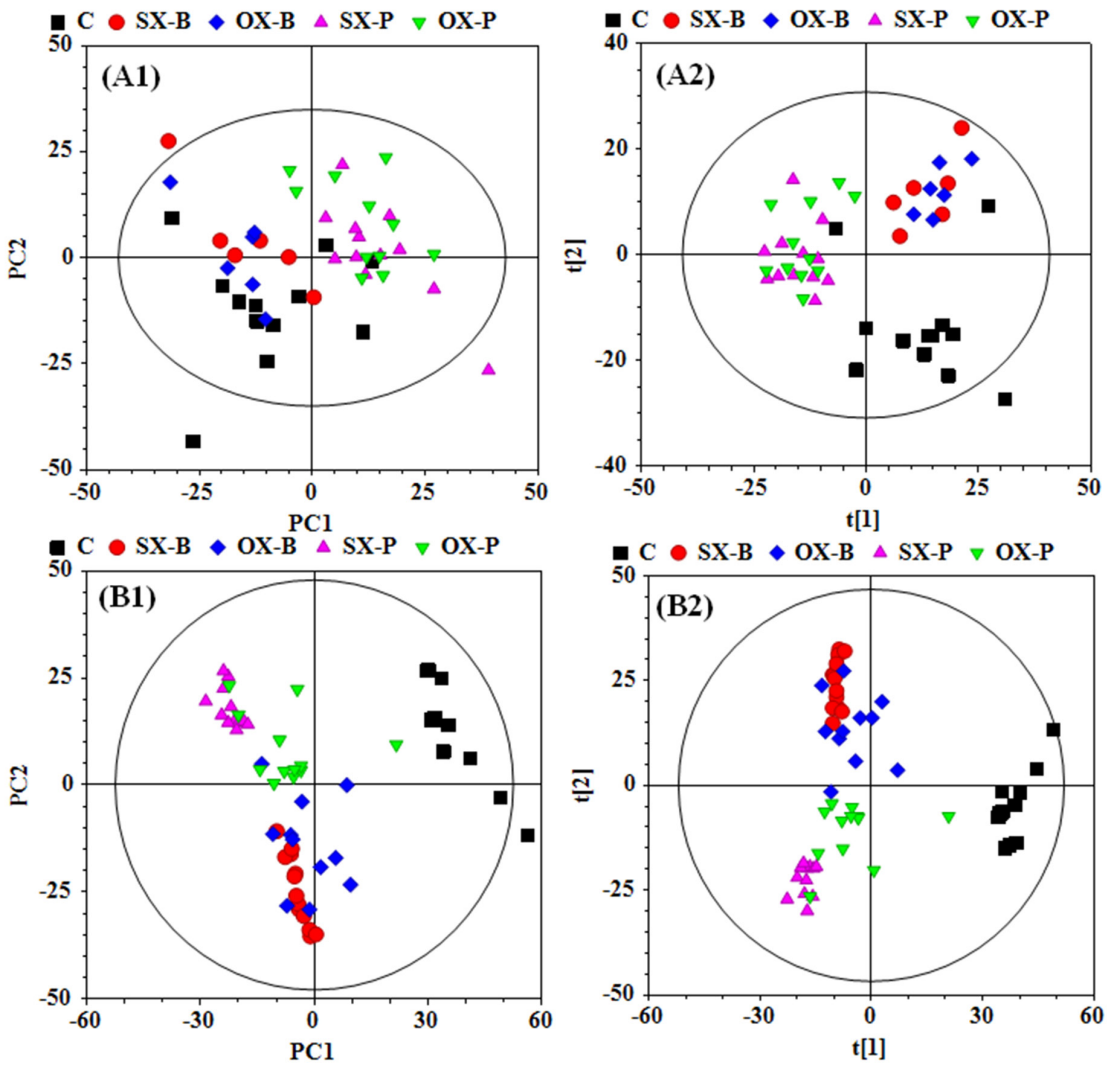
PCA (**A1** and **B1**) and PLS-DA (**A2** and **B2**) scores plots based on the ^1^H NMR data of the serum (**A**) and tissue (**B**) samples from the corresponding mouse groups. C: control groups; -B: BxPC-3 cell induced; -P: Panc-1 cell induced; SX: subcutaneous xenograft group; OX: orthotopic xenograft group.

To investigate the metabonomic difference between SX and OX mouse models of PDAC and identify their metabolic characteristics from the controls, PCA and PLS-DA were performed to give an overview on the NMR data of serum from PDAC groups induced by BxPC-3 and Panc-1 cells and the control mice (Figure 2A1 and 2A2). PCA scores plot displayed the metabolic differences between control and BxPC-3 and Panc-1 cell induced PDAC groups but obvious overlapping between SX and OX groups (Figure 2A1), indicating that the serum metabonomes of SX and OX models were quite similar and difficult to be distinguished. PLS-DA highlighted the distinctive separations between controls and xenograft groups but the evident overlapping between SX and OX groups was still kept, which implied the similar metabolic phenotype between SX and OX groups in serum. The metabonomic similarity in serum between the two models may be due to the fact that serum metabonomes are also the comprehensive reflection from other organs in the body besides the specific tumor-bearing organ (pancreas in this study), that is, the metabolic characteristics of the tumor tissues could be demonstrated in the animal serum wherever the tumor locates. Judging from this aspect, subcutaneous xenograft and orthotopic xenograft models are metabolically equivalent.

In order to identify the metabolic similarity between SX and OX models in serum metabonomes, the pair-comparisons were conducted in the subgroups in both BxPC-3 and Panc-1 groups by using OPLS-DA (Supplementary Figure 1 in the supplemental materials). The undesirable fit and prediction parameters (R^2^ and Q^2^) in the OPLS-DA models demonstrated insignificant differences between SX and OX groups induced by both BxPC-3 and Panc-1 cells, and permutation test of models and the corresponding probability (p-values) via CV-ANOVA also confirmed the metabolic similarity of serum between these two modeling methods.

**Figure 3:**
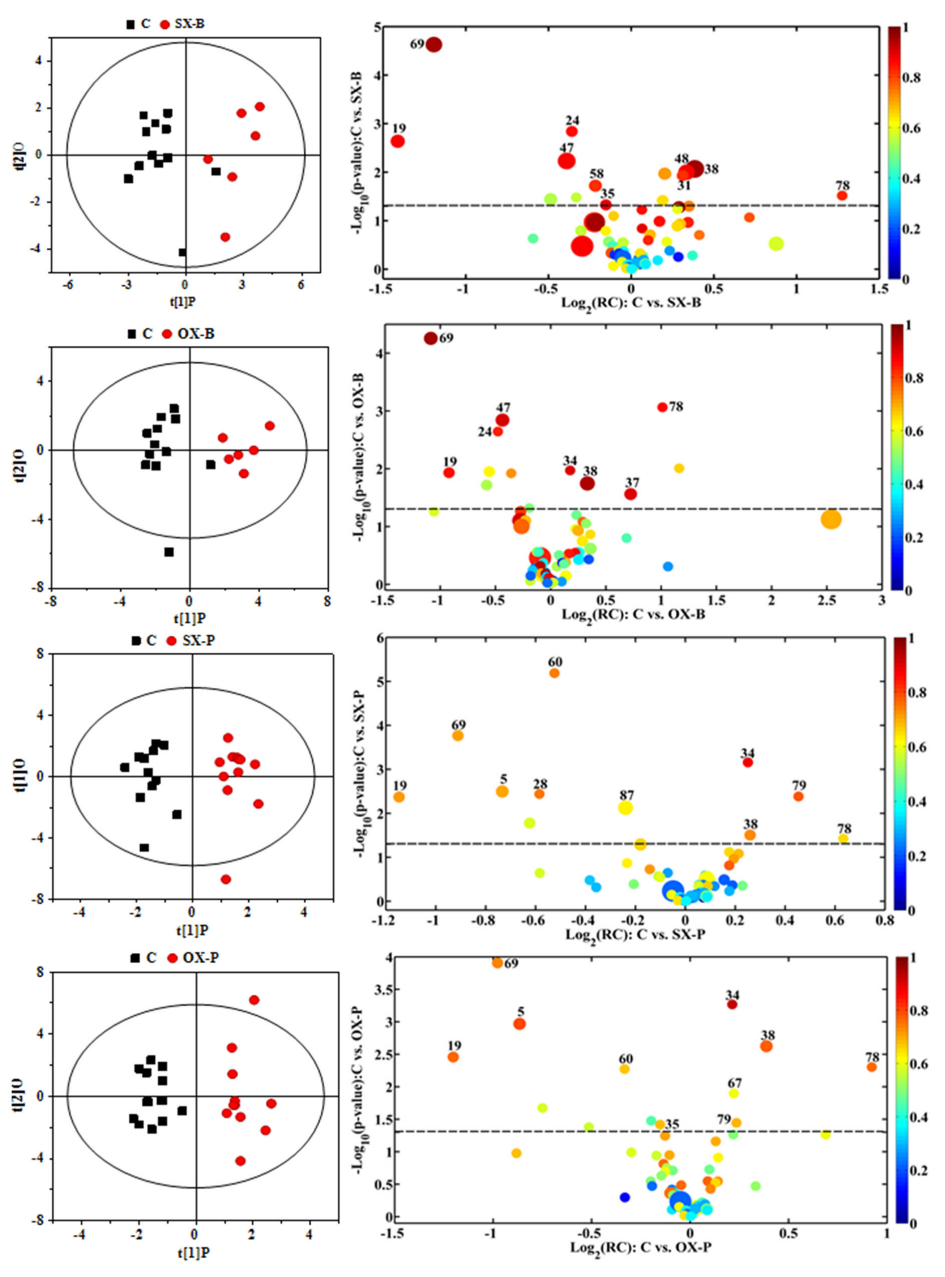
OPLS-DA scores plots (left panels) and the corresponding volcano plots (right panels) derived from ^1^H NMR data of the serum from xenograft groups and their controls C: control groups; B: BxPC-3 cell induced; P: Panc-1 cell induced; SX: subcutaneous xenograft group; OX: orthotopic xenograft group. The marked circles in the color volcano plots represent the metabolites with statistically significant difference. Metabolite numbering is accordance with that as listed in Table 1.

**Figure 4:**
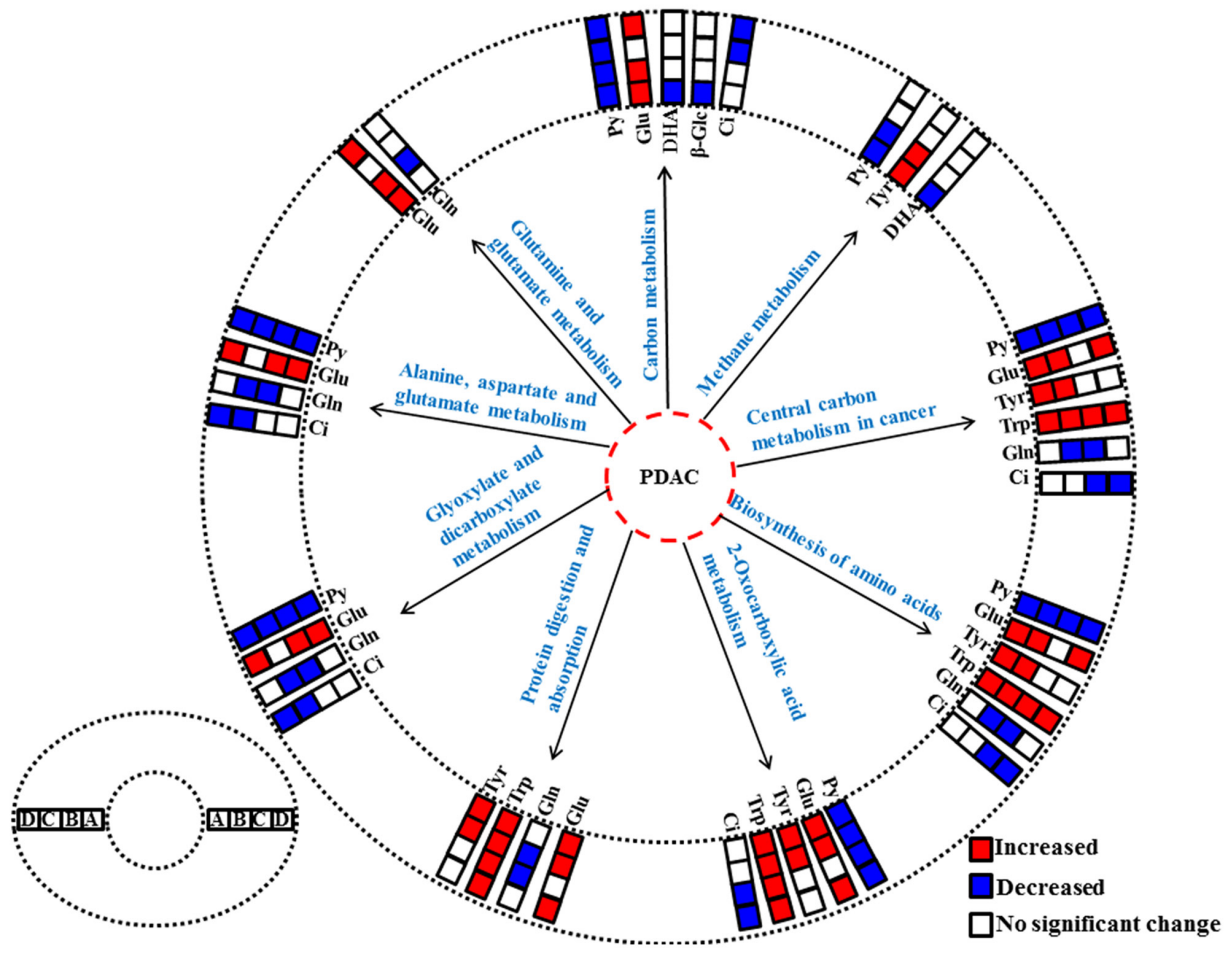
The abnormal metabolic pathways involved in pancreatic ductal adenocarcinoma based on characteristic metabolites in serum metabonomes **(A)**, Panc-1 cell induced subcutaneous xenograft model; **(B)**, Panc-1 cell induced orthotopic xenograft model; **(C)**, BxPC-3 cell induced subcutaneous xenograft model; **(D)**, BxPC-3 cell induced orthotopic xenograft model. Abbreviations for the metabolites are listed in Table 1.

To understand the metabonomic characteristics of SX and OX models and identify the dominantly differential metabolites of the xenograft groups from the controls, the pair-comparisons by using OPLS-DA incorporating Student’s t test were conducted on the NMR data of serum from in BxPC-3 and Panc-1 groups. And the results were visually displayed in volcano plots (Figure 3, where the threshold was defined as p-value of *t-* test less than 0.05 and a certain correlation coefficient |r|>0.755 for BxPC-3 groups and 0.602 for other comparisons), and the corresponding information was listed in Table 2. We noticed that SX and OX models both in BxPC-3 and Panc-1 cell groups demonstrated similar metabonomic characteristics in serum, where the differential metabolites from SX and OX share the same variation trends and no one showed contrary variation trend (Table 2). Based on KEGG analysis, the metabolic pathways involving the dominant metabolites from BxPC-3 and Panc-1 cell induced PDAC groups were similar and most of pathways could be closely associated with the specific mechanism of tumorigenesis and tumor development (Figure 4). The PDAC mainly involved in the abnormal pathway in central carbon metabolism in cancer, metabolism and biosynthesis of amino acids and carboxylic acids, and protein digestion and absorption. As may be expected, no obvious metabonomic difference could be identified in the serum between SX and OX models by comparing the significantly differential metabolites and corresponding pathways. However, the non-significant metabonomic difference in serum may also be due to the limited amount of samples in present study and the specific bioinformation of metabonomic difference should be further investigated to get a deep insight.

**Table 2:** The significantly differential metabolites in serum between xenograft groups and their controls

Metabolites	C-SX-P^a^		C-OX-P		C-SX-B		C-OX-B	
	R^2^X= 32.6% R^2^Y= 0.933 Q^2^= 0.813 p= 1.03×10^−6^		38.9% 0.953 0.866 1.24×10^−7^		37.6% 0.755 0.090 0.858		38.1% 0.837 0.460 0.072	
	RC^b^	R^c^	RC	R	RC	R	RC	R
Tryptophan	0.029	0.656	0.050	0.768	0.45	0.835	0.29	0.874
Tyrosine	0.062	0.784	0.032	0.692	/	/	/	/
N,N-Dimethylglycine	-0.071	-0.747	-0.047	-0.677	/	/	/	/
Dihydroxyacetone	-0.091	-0.757	/	/	/	/	/	/
Glutamate	0.33	0.885	0.27	0.926	/	/	1.35	0.906
Ascorbate	-0.82	-0.720	-0.80	-0.769	-5.92	-0.880	-4.22	-0.862
Pyruvate	-1.08	-0.711	-1.13	-0.744	-10.47	-0.962	-8.80	-0.961
Glycerophosphorylcholine	1.40	0.735	2.23	0.778	15.37	0.966	12.35	0.952
3-Hydroxybutyrate	-2.08	-0.709	-2.20	-0.803	/	/	/	/
β-Glucose	-3.87	-0.633	/	/	/	/	/	/
Phosphocholine	/	/	0.45	0.609	/	/	/	/
Methylmalonate	/	/	/	/	-3.37	-0.826	/	/
Citrate	/	/	/	/	-1.13	-0.862	-1.20	-0.843
Glutamine	/	/	-0.21	-0.684	-1.94	-0.904	/	/
Glycerol	/	/	/	/	/	/	8.48	0.904
LDL	/	/	/	/	-12.23	-0.879	-11.89	-0.912
Ethanolamine	/	/	/	/	1.66	0.825	/	/
VLDL	/	/	/	/	9.45	0.881	/	/

^a^C: control groups; SX-B and OX-B: BxPC-3 cell induced subcutaneous and orthotopic xenograft groups; SX-P and OX-P: Panc-1 cell induced subcutaneous and orthotopic xenograft groups.

^b^RC: relative concentration according to quantified NMR spectroscopy. Positive and negative indicate the metabolites that are more abundant in the xenograft groups or in the control groups, respectively.

^c^R: correlation coefficients. Positive and negative indicate the metabolites that are more abundant in the xenograft groups or in the control groups, respectively. “/” means no significant difference.

### Metabolic characteristics of SX and OX models in tissue metabonomes

To comprehensively assess the metabonomic difference between SX and OX models, PCA and PLS-DA were also performed on the NMR data derived from aqueous extract of BxPC-3 and Panc-1 tissues. Unlike the results in serum, obvious metabonomic distinctions were demonstrated in tissues not only between BxPC-3, Panc-1 and the control groups but also between SX and OX groups in their PCA scores plot (Figure 2B1), and PLS-DA highlighted their distinctive separations (Figure 2B2). Furthermore, the obvious metabonomic differences were also observed between BxPC-3 and Panc-1 groups in the tissue metabonomes. It indicated that the model (SX and OX)- and cell strain (BxPC-3 and Panc-1)-specific metabonomic characteristics were induced in tissue.

**Figure 5:**
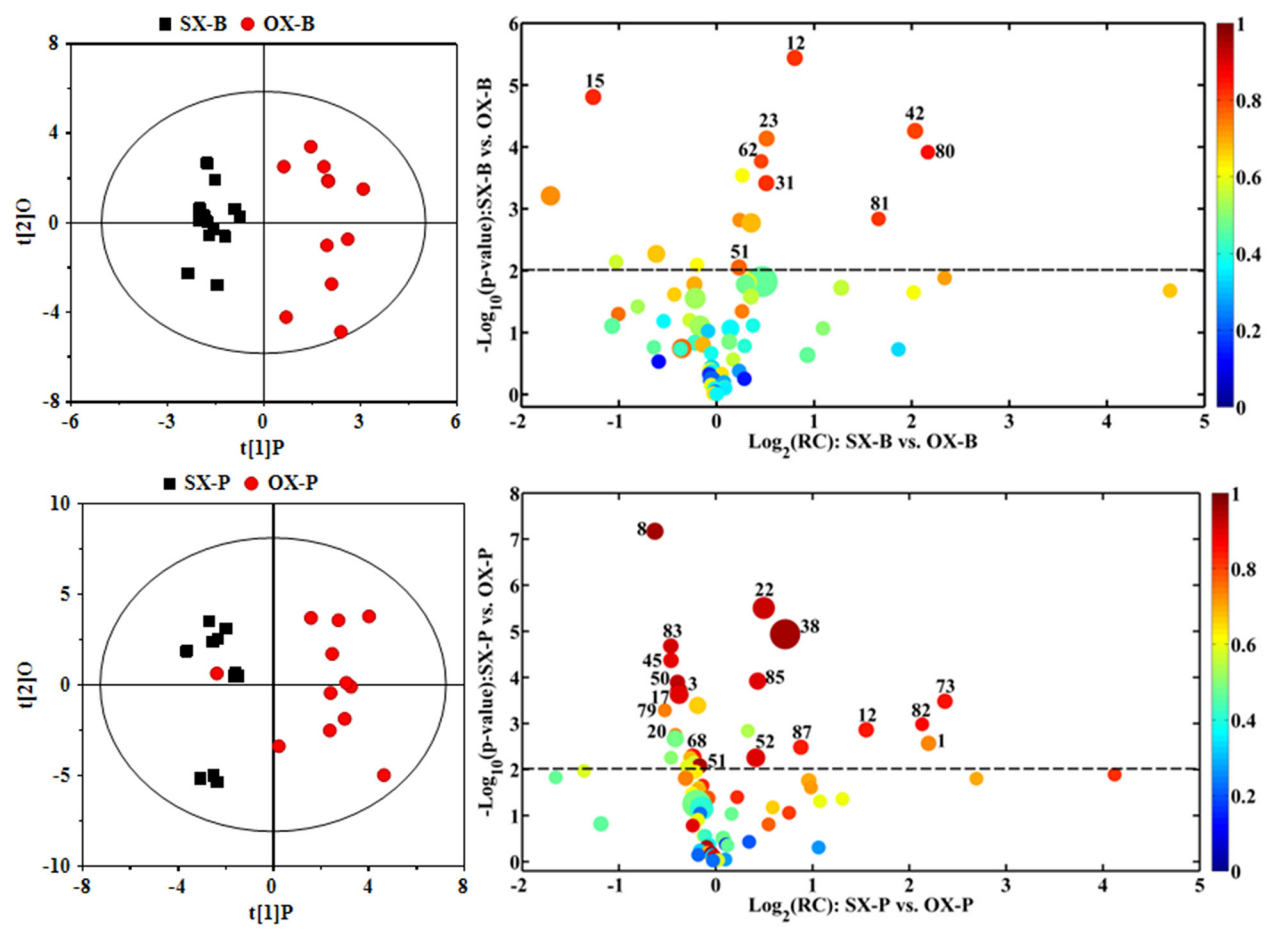
OPLS-DA scores plots (left panels) and the corresponding volcano plots (right panels) derived from ^1^H NMR data of the tissue samples from BxPC-3 (-B) and Panc-1 (-P) cell induced subcutaneous(SX-) and orthotopic (OX-)xenograft groups The marked circles in the color volcano plots represent the metabolites with statistically significant difference. Metabolite numbering is accordance with that as listed in Table 1.

**Figure 6:**
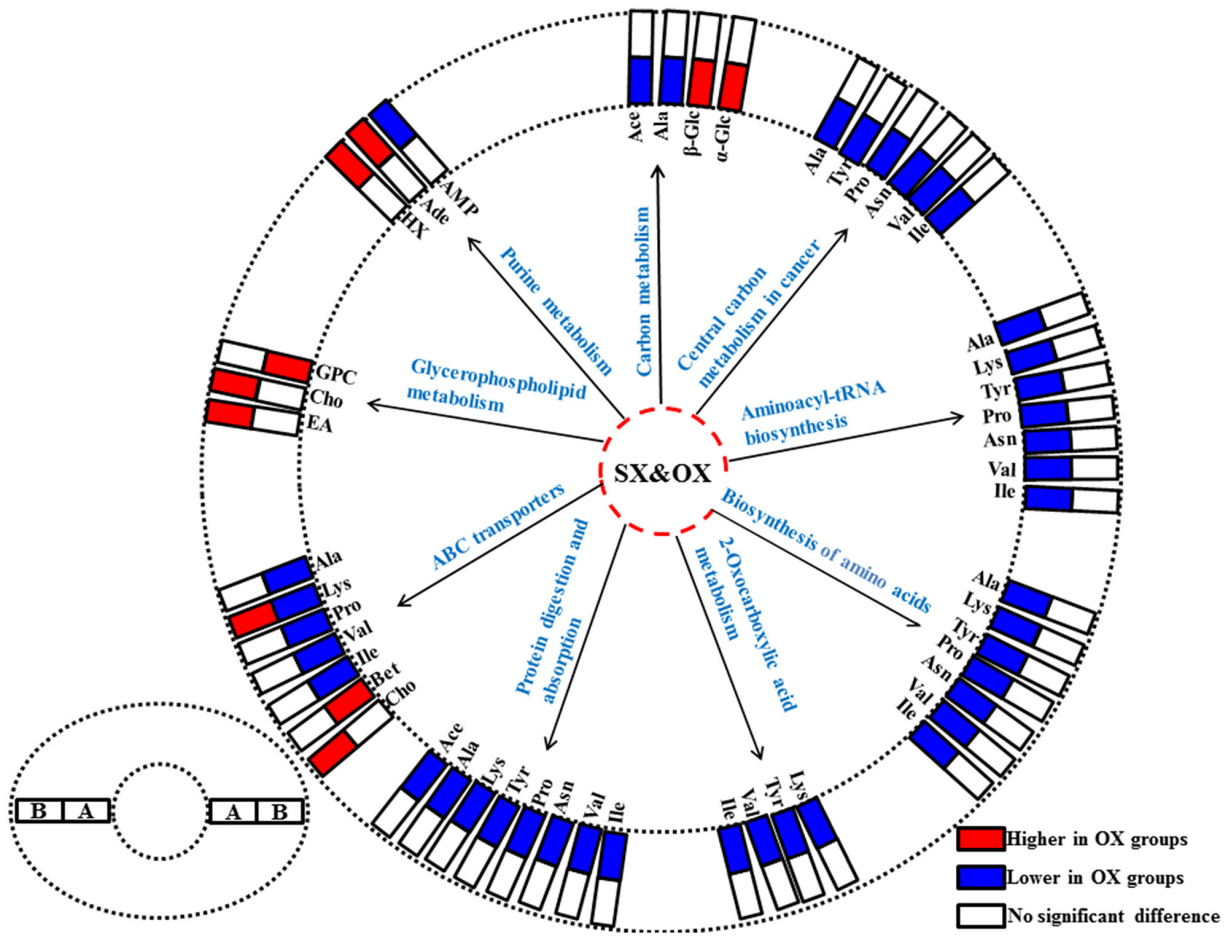
The differentiated metabolic pathways between subcutaneous and orthotopic xenograft groups based on the dominant metabolites in tissue metabonomes The pair-comparisons: **(A)**, subcutaneous and orthotopic xenograft groups induced by Panc-1 cell; **(B)**, subcutaneous and orthotopic xenograft groups induced by BxPC-3 cell. Abbreviations for the metabolites are listed in Table 1.

In order to understand the detailed metabolic differences between SX and OX models and identify the differential metabolites in tumor tissues, OPLS-DA was performed on their NMR data from both BxPC-3 and Panc-1 cells induced PDAC groups (Figure 5). With the favorable model parameters R^2^ and Q^2^ (Table 3), the pattern recognition models can distinguish the tumor tissue from SX and OX model with considerable validity and efficiency, and the permutation plots (Supplementary Figure 2) and p-values via CV-ANOVA also confirmed the obvious metabolic differences between SX and OX groups. The metabolites, which had statistically significant difference in t-test (p<0.01) and correlation coefficients more than 0.735, were considered to be most valuable components for further investigation (Table 3). Based on the comparison of SX and OX in BxPC-3 groups, the higher levels of nicotinamide, uracil, uridine, lysine, adenosine, hypoxanthine, ethanolamine, choline and lower levels of adenosine monophosphate in OX tissues dominantly contributed to the metabonomic difference between them. Meanwhile, in Panc-1 groups, the lower levels of asparagine, tyrosine, lysine, valine, isoleucine, leucine, 2-hydroxybutyrate, acetate, proline and alanine accompanied by the higher levels of uridine diphosphate glucose, surcose, adenosine, 1-methylhistidine, β-glucose, α-glucose, malate and GPC in OX tissues were also identified as the dominantly differential metabolites. In terms of pathways (Figure 6), in the BxPC-3 groups, ABC-transporter, glycerophospholipid and purine metabolism were identified as discriminatory cancer-associated metabolic pathway between SX and OX models while more pathways, including central carbon metabolism in cancer, biosynthesis of aminoacyl-tRNA and amino acids, 2-oxocarboxylic acid metabolism and protein digestion and absorption, were identified in Panc-1 groups. According to these discriminatory metabolites and metabolic pathways, the metabolic discrimination between SX and OX models concentrated on lipid and nucleotide metabolisms in Bxpc-3 group, and energy, lipid, nucleotide and amino acid metabolisms in Panc-1 group.

**Table 3:** The significantly differential metabolites in tissues between subcutaneous and orthotopic xenograft groups

Metabolites	SX-P&OX-P^a^		SX-B&OX-B^a^	
	R^2^X=55.9%, R^2^Y=0.749, Q^2^=0.632, p=5.21×10^−4^		R^2^X=32.6%, R^2^Y=0.902, Q^2^=0.643, p=3.96×10^−4^	
	RC^b^	R^c^	RC	R
Nicotinamide	/	/	0.36	0.807
Uracil	/	/	1.46	0.863
Uridine	/	/	1.80	0.823
Lysine	-0.50	-0.953	2.55	0.774
Adenosine	0.74	0.848	2.59	0.822
Hypoxanthine	/	/	3.34	0.804
Adenosine monophosphate	/	/	-4.08	-0.833
Ethanolamine	/	/	4.64	0.825
Choline	/	/	5.24	0.766
Uridinediphosphate glucose	0.19	0.868	/	/
Asparagine	-0.26	-0.745	/	/
Tyrosine	-0.28	-0.749	/	/
Valine	-0.53	-0.915	/	/
Surcose	0.58	0.857	/	/
Isoleucine	-0.69	-0.897	/	/
Leucine	-0.86	-0.931	/	/
1-Methylhistidine	0.87	0.739	/	/
β-Glucose	0.90	0.846	/	/
α-Glucose	1.30	0.905	/	/
2-Hydroxybutyrate	-1.54	-0.942	/	/
Acetate	-1.56	-0.961	/	/
Proline	-1.62	-0.859	/	/
Malate	2.14	0.903	/	/
Alanine	-2.18	-0.899	/	/
Betaine	3.33	0.923	/	/
Glycerophosphocholine	6.74	0.964	/	/

^a^SX-B and OX-B: BxPC-3 cell induced subcutaneous and orthotopic xenograft groups; SX-P and OX-P: Panc-1 cell induced subcutaneous and orthotopic xenograft groups.

^b^RC: relative concentration according to quantified NMR spectroscopy. Positive and negative indicate the metabolites that are more abundant in the orthotopic and subcutaneous xenograft groups, respectively.

^c^R: correlation coefficients. Positive and negative indicate the metabolites that are more abundant in the orthotopic and subcutaneous xenograft groups, respectively. “/” means no significant difference.

## DISCUSSION

In this study, in order to be more convincing of metabonomic comparison, we chose Panc-1 and BxPC-3 cell strains to establish the experimental groups. With common suppressor genes such as P53 and P16, Panc-1 have a mutant Kras gene (Kras^G12D^) while BxPC-3 have a wild type Kras gene [10], which related with several crucial metabolisms for PDAC growth [11, 12]. Thus, these models could cover most of metabolic characteristics of PDAC strains used in scientific researches, providing a comprehensive assessment of metabonomic discrimination between SX and OX models. The average length, width and volume of tumors (calculated based on universal formula: length*width*width/2) in SX-B was 8.63±2.27 mm, 6.94±1.26 mm and 219.92 mm^3^, respectively. The average length, width and volume of tumors in OX-B was 7.35±1.19 mm, 6.53±0.98 mm and 166.92 mm^3^, respectively. For Panc-1, these parameters were 7.80±2.01 mm, 6.92±0.94 mm, 196.44±91.80 mm^3^ in SX-P and 7.34±1.41, 6.20±0.95, 154.44±74.25 mm^3^. No significant differences were observed for these pair-comparing parameters from SX and OX models. Therefore, the metabonomic difference between SX and OX models may not be attributed to the difference in size of tumors. In our study, in order to eliminate metabolic impact from the methodological difference of operations between OX and SX models, all groups were operated with almost the same surgical operations. Although these procedures in our study differ with conventional modeling methods, the perfect match of all groups could provide more precise metabonomic discrimination only caused by the methodological difference.

According to results of multivariate statistical analyses, the metabonomic differences were indistinctive in serum but significant in tissue, therefore we focus on the bioinformation contained in metabonomic difference of tissue between OX and SX models.

ABC-transporters are a large family of transmembrane proteins transporting nutrients into cells, and their abnormality has been associated with many diseases and cancers [13, 14]. Furthermore, previous literatures have demonstrated that ABC-transporters play an important role not only in chemoresistance of PDAC [15] but also connect with the initiation and progression of PDAC, further served as predictors for poor prognosis [16, 17]. As the unique role of ABC-transporters in PDAC oncobiology, the difference of ABC-transporters metabolism may partly account for biobehavioral discriminations between SX and OX models such as ascites generation, lymph node and liver metastases. It also implied that OX and SX models of PDAC would be unequivalent for pharmaceutical and clinical trials for chemical agents.

According to previous studies, the alteration of glycerophospholipid metabolism, characterized by increase of choline and phosphocholine (PC), is a critical aspect of tumor metabolism [18, 19, 20, 21]]. Besides generating more substrates for cell proliferation, the glycerophosphodiesterase (EDI3)-mediated choline glycerophospholipid metabolism can also control tumor cell migration via PKC signaling and module cellular signaling through down-stream products [22]. However, the changes of the specific metabolites involved in glycerophospholipid metabolism including GPC, PC and choline are not quite consistent in the previous studies on PDAC [23], which may be due to the differences in experimental models and comparison objectives. The contradictory results confuse us to give a specific elucidation about our finding. However, the difference of choline and GPC concentration between SX and OX models both in Bxpc-3 and Panc-1 groups strongly indicated that SX and OX models were unequivalent in the tumor-associated glycerophospholipid metabolism.

As one of the major component in nucleotide metabolism, purine metabolism was highly disturbed in tumor metabolism. In tumor cells, the pyruvate kinase M2 is often in less active form [24, 25, 26], followed by up-stream substrates of pyruvate accumulation and enhanced synthesis of ribose-5-phosphate and nucleic acid [27]. In addition, purine metabolism plays key roles in cell signaling and tumor immunology [28]. In extracellular microenvironment, ATP and its sequential hydrolyzed product, adenosine, can act on nucleotide receptors P2 and adenosine receptors P1 respectively, which are expressed by cancer cell and infiltrating immune cells. Depending on different condition, ATP and adenosine can promote through direct activation and immunosuppression, or inhibit cancer growth through immunoactivation [29, 30, 31, 32]]. Thus, the purinergic signaling pathways were significantly different between SX and OX models, which were indicated by the difference of adenosine and AMP concentration in our study. This difference may lead to erroneous assessment to chemotherapeutic efficacy of specific anticancer agents such as mitoxantrone and oxaliplatin which owe their anticancer effects to the ability of triggering an excessive anticancer immune response [33].

The difference of carbon metabolism between OX and SX models could definitely reflect the heterogeneity of many enzymes and receptors targeted as the potential chemotherapeutic sites, which may result in totally different outcome of pharmaceutical and clinical trials [34]. In our study, the metabolic differences of amino acids involved in carbon metabolism were quite notable between SX and OX models in Panc-1 group. Relative to SX model, the consistent low level of branched chain amino acids and tyrosine in OX model tissue implied that the transporting function of amino acids was significantly decreased, which may related with the L-type amino acid transporter (LAT1), the most common transporter for essential amino acids delivery. By now, several researches have demonstrated that overexpression of LAT1 can stimulate cancer growth via mTOR pathways and be closely related with poor prognosis of several cancers [35, 36, 37]. In return, depressing LAT1 can also inhibit the tumor cell growth [38]. In addition, the low level of alanine, lysine, asparagine and proline in OX models may be due to a relatively weak ability to synthesize and uptake non-essential amino acids (NEAAs), or abundant consuming caused by rapidly proliferation of tumor. Nowadays, NEAAs are explicitly identified as key players in development of tumor including PDAC [39]. For instance, proline plays a special role in linkage between the metabolism and epigenetics through modulating intermediates of epigenetic regulation [40]. Furthermore, a recent research uncovered a unique role of autophagic alanine secretion in energy metabolism through tricarboxylic acid cycle (TCA) efflux in PDAC, which may even outcompete glycolysis and glutaminolysis [41]. However, the specific mechanism causing metabolic difference between SX and OX models is yet to determine and further investigation will be needed to get a deep insight.

The particular reasons for metabolic difference between SX and OX models still remain unclear. However, since the main methodological difference between these models is the position of tumor implantation, we hypothesize that the tumor microenvironment may give the most important contribution to the metabonomic difference. As demonstrated by previous researches, the tumor microenvironment could impose a great influence on the fibrosis, invasion and metastasis of pancreatic cancer [42]. As the predominant cell type of tumor microenvironment, pancreatic stellate cells (PaSCs) play a key role in the intimate interaction between PDAC and tumor microenvironment [43, 44]. Recently, an important metabolic pathway was uncovered in PDAC. *In vitro*, PaSCs could secrete alanine through autophagy to fuel the *Krebs cycle* rather than glycolysis to support tumor metabolism [41]. The intermediates of TCA were not only depleted through oxidative phosphorylation to generate ATP but also acted as building blocks for biosynthesis of fatty acid and NEAAs. Thus, although the role in tumor metabolism of PDAC has yet to be completely understood, the existence of PaSCs could undoubtedly trigger significant metabolic difference between OX and SX models. However, this hypothesis may need further research to be confirmed and improved.

Our study demonstrated that SX models are not equivalent to OX ones. Although subcutaneous transplantation offers a simple, inexpensive, reproducible and quantifiable advantage, it displayed its inherent limitations, i. e. it does not accord with the biological behaviors and tumor microenvironment of PDAC. And orthotopic implantation may be more favorable to simulate PDAC in patients due to their tumorigenesis similarity.

## CONCLUSION

In this pancreatic cancer-related study, an obvious metabonomic difference was demonstrated between SX and OX animal models in tissue samples by using NMR-based strategy. The metabonomic differences were mainly associated with ABC transporters, glycerophospholipid metabolism, purine metabolism and central carbon metabolism in cancer and may be attributed to the difference of tumor microenvironment caused by different location of implantation. These findings indicated that the implantation of PDAC cell subcutaneously or in other organ cannot simulate the pathophysiological and system-biological features of PDAC as well as the OX models. OX animal models should be more favorable for cancer involved scientific researches, especially for the pharmacodynamical and pathophysiological experiments.

## MATERIALS AND METHODS

### Cell culture and animal modeling

Panc-1 and BxPC-3 cell strains were purchased from Shanghai Institute of Cell Biology and incubated with RPMI1640 supplemented with 10% fetal bovine serum (Gibco, Thermo Fisher Scientific, Shanghai, China) in cell incubator (3110, Thermo Scientific) with a circumstance of 37 °C and 5% CO_2_. BALB/c nude mice (male, 4-6 weeks, weighing 20-24 g) (NO: SCXK (HU) 2012-0002) were purchased from Slac laboratory animals Co., Ltd (Shanghai, China) and fed for 1 weeks under a standard laboratory conditions in Fujian Medical University Animals Center (Fuzhou, china) for acclimatization before operations.

The protocol of this study was in accordance with the principles of Guide for the Care and Use of Laboratory Animals [45] and approved by the ethical committee of Fujian Medical University Union Hospital. After a three-day cell culture, the culture flasks of Panc-1 and BxPC-3 cells were digested with 0.125% trypsogen (Gibco, Thermo Fisher Scientific, Shanghai, China), followed by washing for three times with phosphate buffered saline (PBS, Hyclone, GE Healthcare, Logan, Utah, US), and consequently resuspended in PBS (1×10^6^ cells suspended in 100 μL of PBS). Then, the cell suspensions of Panc-1 and BxPC-3 were subcutaneously injected into the right axilla of a mouse respectively. After a month of feeding, the subcutaneous tumors were harvested and disintegrated into 1-mm^3^ particles for transplantation.

The operation of OX-P and OX-B groups started with a horizontal incision inferior to left costal margin and exposed the body and tail of pancreas. Then, one Panc-1 or BxPC-3 particle was implanted on the surface of pancreas for each mouse and fixed with 1 μL of albumin glue (Compont, Compont pharmaceutical Science & Technology Co. Ltd., Beijing, China). Meanwhile, 1 mL of saline was injected subcutaneously into the back of mice. Reversely, the SX-P and SX-B groups were injected subcutaneously into the back of the mice with a Panc-1 or BxPC-3 particle by using a 16-gauge syringe, respectively, and followed by a surgical exploration of pancreas with a fixation of 1 μL albumin glue but without particle implantation. In present study, 12 and 15 BALB/c mice were prepared for establishment of SX and OX models respectively (See the representative images of successful establishment of models as displayed in Supplementary Figure 3A-C in the supplemental materials). For Bxpc-3 cell strain, the successful percentage of SX models was 100% (12/12) while 73.33% (11/15) in OX models; For Panc-1 cell strain, the successful percentage of SX models was 91.67% (11/12) while 80% (12/15) in OX models. To be completely comparative, the control group (n=12) was operated with subcutaneously injection of saline and surgical exploration of pancreas without cell implantation.

### Sample preparation and NMR spectroscopy for serum and tissue from mice

Four weeks after operations, all mice were sacrificed under continuous airway-anesthesia for sample collection. As described in our previous study [46], before mice’s execution, at least 800 μL of blood was collected in 1.5-mL EP tube with a 30-60 min standing, followed by a 10-min centrifugation at 2,000 g for serum preparation. After blood collection, the tumor tissues were immediately collected. To eliminate the contamination of these tissues, the tumors were disposed with *en bloc* resection. Then, the infiltrated and adherent tissues around the tumor including tumor capsular, pancreas, skin and muscle were entirely stripped until leaving pure tumor tissues characterized with a homogeneous, hoary and solid mass. Sequentially, these tumor tissues were rapidly divided into two parts, one for histological analysis (Supplementary Figure 3D-I in the supplemental materials) and another one for ^1^H NMR analyses. All samples were snap frozen with liquid nitrogen and stored at -80 °C for NMR spectroscopy. Unfortunately, some biosamples were not available due to the misconducted storing methods. Finally, there are only 6 serum samples remaining in SX-B and OX-B groups. Tissue were extracted with conventional methanol-chloroform extraction method as described in our previous report [47], and the serum and tissue samples were prepared accordingly for NMR detection.

All of the serum and tissue samples were analyzed randomly on a Bruker Ascend^TM^ NMR spectrometer (Bruker Corporation, Karlsruhe, Germany) at 600.13 MHz proton frequency and 295 K. For serum samples, ^1^H NMR spectra were acquired by using the water-suppressed Carr-Purcell-Meiboom-Gill (CPMG, [RD-90°-(τ-180°-τ)_n_-ACQ]) pulse sequence with a spectral width of 12 KHz, an acquisition time of 2.73 s, a relaxation delay of 4 s, a scan accumulation of 16 times, and a data point of 32 K. For tissue samples, the detection was performed with the pulse sequence of nuclear Overhauser effect spectroscopy plus water suppression (NOESYPR1D, [RD-90°-*t*_1_-90°-*t*_m_-90°-ACQ]) with a spectral width of 12 KHz, an acquisition time of 2.66 s, a relaxation delay of 4 s, a scan accumulation of 16 times, t_1_ of 4 μs, a data point of 32 K and a mixing time of 100 ms.

### NMR spectral processing

All free induction decays (FIDs) were multiplied by an exponential weighting function equivalent to a line-broadening of 0.3 Hz for tissue samples and 0.5 Hz for serum samples to increase the signal-to-noise ratio. Then, by using MestReNova (V9.0, Mestrelab Research, Santiago de Compostela, Galicia, Spain), all spectra were deposed with Fourier transformation and manual corrections for phase and baseline. In serum samples, the chemical shift was referenced to the double peaks of endogenous lactate at δ1.33, and the spectral regions of δ4.67-δ5.22 and δ5.70-δ6.00 were removed to eliminate the interference of residual aquatic and ureal signals. The remainder spectral regions (δ0.6-8.6) were integrally segmented into discrete regions of 0.002 ppm. In tissue samples, the chemical shift was referenced to TSP at δ0.00 and the spectral regions of δ4.7-5.15 and δ3.34-3.39 were eliminated for aforementioned reasons, followed by segmentation with 0.004 ppm interval in spectral region of δ0.7-10.0. All the disposed data were normalized and homogenized for multivariate statistical analysis.

### Univariate and multivariate statistical analyses

To get insight into the bioinformation contained in NMR spectra, all processed spectral data were analyzed by using multivariate statistical analysis with assistance of SIMCA-P+ (V14.0, Umetrics AB, Umea, Sweden). These analyses could simplify the complex information into several dominant components to demonstrate explicitly the metabonomic difference between groups. To give an overview of metabonomic profile from control, principal component analysis (PCA) and partial least squares discriminant analysis (PLS-DA) were conducted with mean center scaling and unit variance scaling, respectively. Then, for better understanding of the specific metabonomic difference between all subgroups in BxPC-3 and Panc-1 groups, the pair comparisons were performed and validated with 10-fold cross validation and permutation test with the first two principal components including a predictive component and an orthogonal component by using PLS-DA with orthogonal signal correction (OPLS-DA) with Parato scaling. The parameter R^2^ and Q^2^, derived from cross validation and permutation test, represented the degree of modeling fitting and the predictive ability, and the p-values from CV-ANOVA demonstrated the significance of the metabolic differences, thus to be critical for validation of statistical analyses and pattern recognition.

The metabolites contributed to the metabonomic difference in pair-comparisons were marked and color-coded based on the corresponding correlation coefficients in OPLS-DA loading volcano plots, where a hot color corresponds to the significant difference between classes while a cool color corresponds to no significant differences. In addition, the relative concentrations of metabolites through calculating the integral area of the corresponding signals were compared and statistical analyzed with Student’s t test for better reliability of characteristic metabolites’ screening, which represented as the X (indicated as −log_2_ of fold changes in concentration of differential metabolites) and Y (indicated as −log_10_ of *t*-test statistically *p*-values) axis in the loading volcano plots, respectively. The *p*-values for statistically significant difference were set as less than 0.05 for serum comparison and 0.01 for tissue comparison, respectively. Positive log_2_ value of fold changes indicate higher concentration of metabolites in the xenograft groups than in controls (for the serum comparison) or in subcutaneous xenograft groups (for the tissue comparison); negative values indicate higher concentrations of metabolites in control (for the serum comparison) or subcutaneous xenograft (for the tissue comparison) groups than in the xenograft groups.

To demonstrate the weight of metabolites in metabonomic difference in pair-comparisons, the visualized circles whose ranges were positively correlated with the concentrations (the radius was indicated as 15+2*concentration ratio), were also drew in the loading plots. Each circle in the volcano plots represents one metabolite, and the circle is colored according to the correlation coefficient of |r| and their cutoff values for the statistical significance were based on the discrimination significance at the level of p < 0.05 and the corresponding degree of freedom in the pair-wise comparison. Thus, the loading plots could demonstrate integrated information about metabonomic difference between groups, fully contributed to further metabolic pathways analysis.

### Metabolic pathways analysis

To get a deep insight into the metabolic pathways involved in the metabonomic difference between SX and OX models, a comprehensive pathway analysis by using KEGG and MBROLE online service was performed on the differential metabolites derived from comparison of SX-OX models in both BxPC-3 and Panc-1 groups [48, 49].

### Additional information

Supplemental materials contain the OPLS-DA and the validation for serum and tissue profiles between subcutaneous and orthotopic xenograft groups.

## SUPPLEMENTARY MATERIALS FIGURES


