# Supplementary material for: Identification and causes of metabonomic difference between orthotopic and subcutaneous xenograft of pancreatic cancer
Source: Oncotarget. 2017 May 22;8(37):61264–81. doi: 10.18632/oncotarget.18057 (PMC5617422; doi:10.18632/oncotarget.18057)
Supplement: Supplementary file 1 [file oncotarget-08-61264-s001.pdf]

Identification and causes of metabonomic difference between orthotopic and subcutaneous xenograft of pancreatic cancer

SUPPLEMENTARY MATERIALS

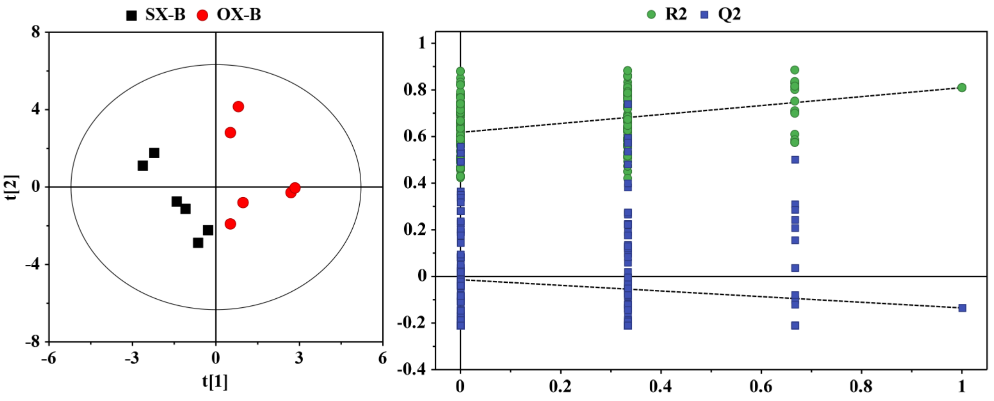

R^2X=43.2%, R^2Y=0.810, Q^2=-0.135, p>0.99

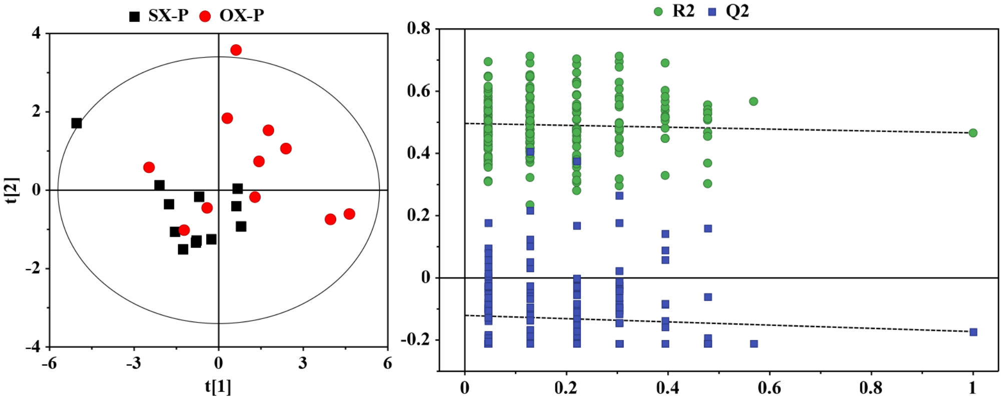

R^2X=38.1%, R^2Y=0.466, Q^2=-0.173, p>0.99

Supplementary Figure 1: OPLS-DA scores plots (left panels), the corresponding permutation plots (n=200) (right panels) and the results from CV-ANOVA (the p values) for serum profiles between subcutaneous xenograft (SX) and orthotopic xenograft (OX) groups induced by BxPC-3 cell strain (B) and Panc-1 cell strain (P).

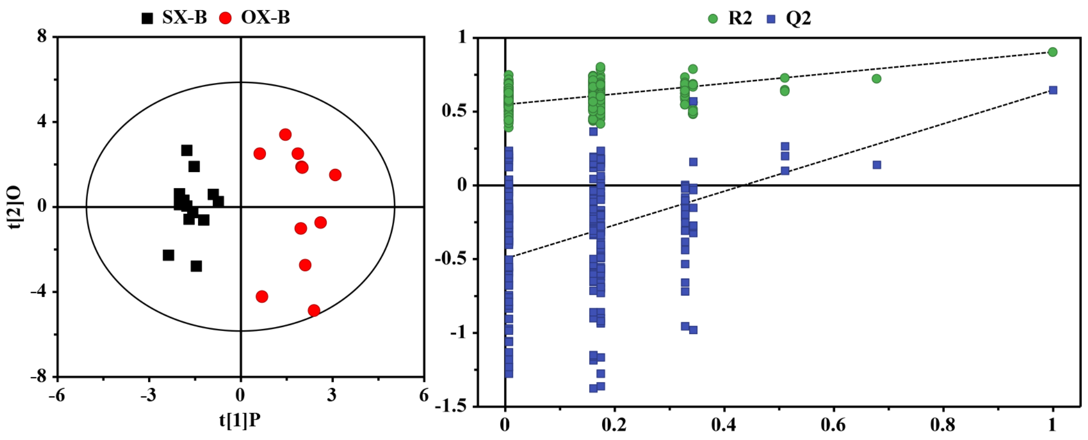

R^2X=32.6%, R^2Y=0.902, Q^2=0.643, p<0.001

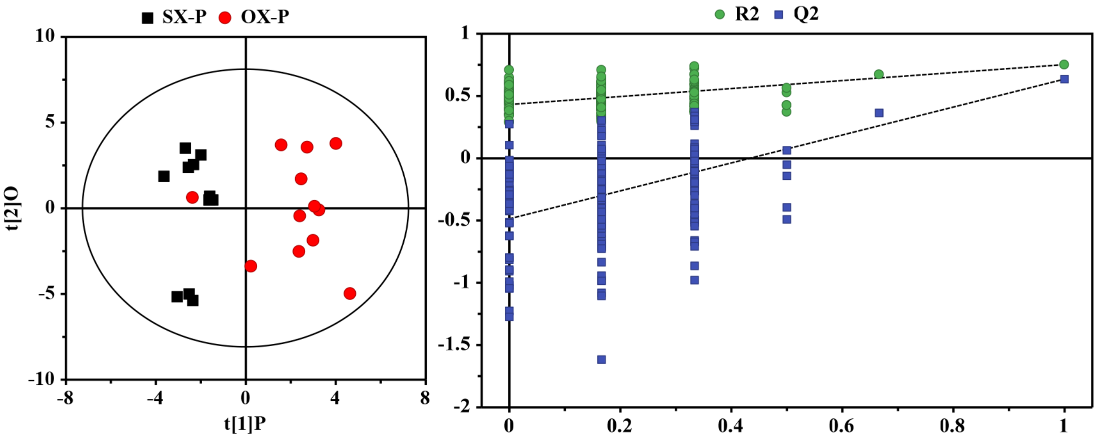

R^2X=55.9%, R^2Y=0.749, Q^2=0.632, p<0.001

Supplementary Figure 2: OPLS-DA scores plots (left panels), the corresponding permutation plots (n=200) (right panels) and the results from CV-ANOVA (the p values) for tissue profiles between subcutaneous xenograft (SX) and orthotopic xenograft (OX) groups induced by BxPC-3 cell strain (B) and Panc-1 cell strain (P).

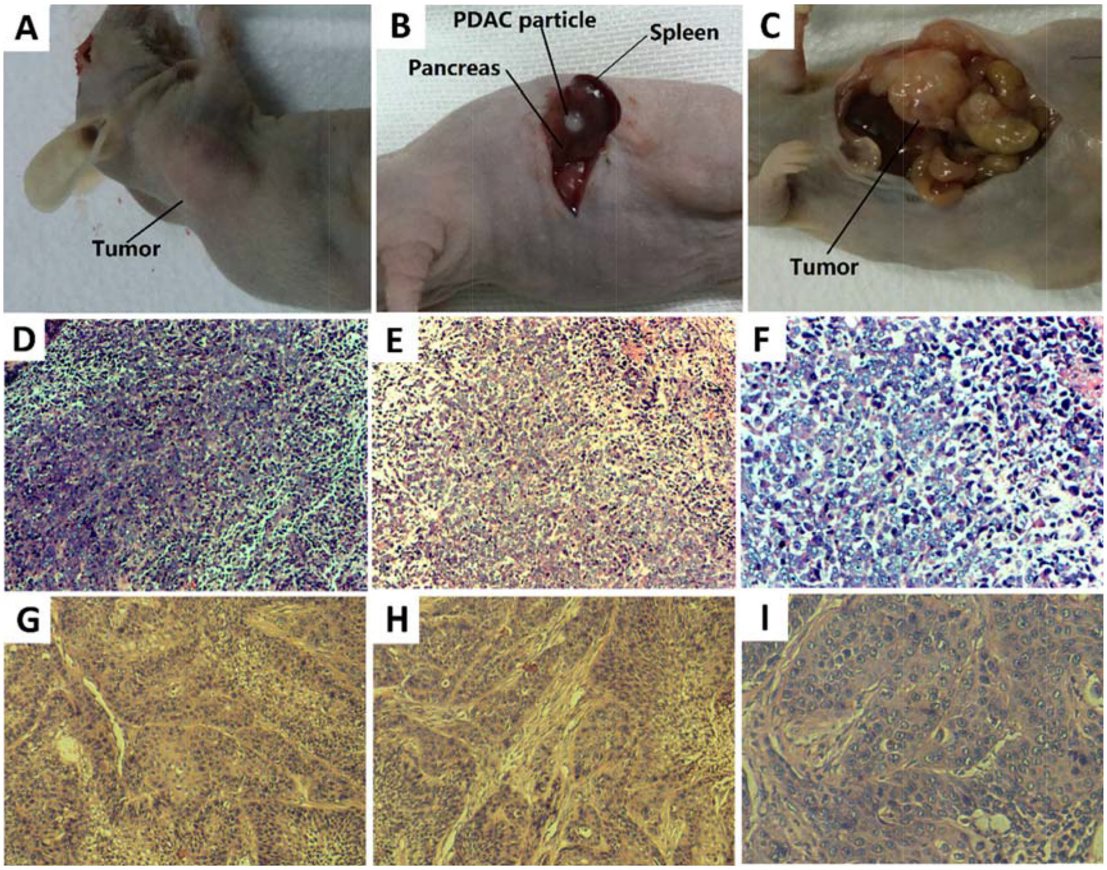

Supplementary Figure 3: The representative images of subcutaneous xenograft (SX) and orthotopic xenograft (OX) models induced by BxPC-3 cell strain (B) and Panc-1 cell strain (P) and corresponding histological observation. **(A)** the representative images of successful establishment of SX models, four weeks after operation. **(B)** The diagrammatic drawing of implantation of PDAC particles on the body and tail of pancreas. **(C)** The representative images of successful establishment of OX models. **(D)** (100 fold, H&E staining) the histological observation of tumor tissue derived from SX-P. **(E)** (100 fold, H&E staining) the histological observation of tumor tissue derived from OX-P. **(F)** (200 fold, H&E staining) the histological observation of tumor tissue derived from OX-P. **(G)** (100 fold, H&E staining) the histological observation of tumor tissue derived from SX-B. **(H)** (100 fold, H&E staining) the histological observation of tumor tissue derived from OX-B. **(I)** (200 fold, H&E staining) the histological observation of tumor tissue derived from OX-P. Images D-F and G-I confirmed that typical undifferentiated and moderate to well differentiated tumors were successfully established, respectively.
